# Universal Features for the Classification of Coding and Non-coding DNA Sequences

**DOI:** 10.4137/bbi.s2236

**Published:** 2009-06-03

**Authors:** Nicolas Carels, Ramon Vidal, Diego Frías

**Affiliations:** 1 Fundação Oswaldo Cruz (FIOCRUZ), Instituto Oswaldo Cruz (IOC), Laboratório de Genômica Funcional e Bioinformática, Rio de Janeiro, RJ, Brazil; 2 Universidade Estadual de Santa Cruz (UESC), Núcleo de Biologia Computacional e Gestão de Informações Biotecnológicas, Ilhéus, BA, Brazil. Email: nicolas.carels@gmail.com

**Keywords:** genomics, exon prediction, purine bias, coding features, open reading frame, ancestral codon

## Abstract

In this report, we revisited simple features that allow the classification of coding sequences (CDS) from non-coding DNA. The spectrum of codon usage of our sequence sample is large and suggests that these features are universal. The features that we investigated combine (i) the stop codon distribution, (ii) the product of purine probabilities in the three positions of nucleotide triplets, (iii) the product of Cytosine, Guanine, Adenine probabilities in 1st, 2nd, 3rd position of triplets, respectively, (iv) the product of G and C probabilities in 1st and 2nd position of triplets. These features are a natural consequence of the physico-chemical properties of proteins and their combination is successful in classifying CDS and non-coding DNA (introns) with a success rate >95% above 350 bp. The coding strand and coding frame are implicitly deduced when the sequences are classified as coding.

## Introduction

Since amino acids are encoded by codons, which are triplets of nucleotides (A, C, G or T, i.e. Adenine, Cytosine, Guanine and Thymine, respectively),coding DNA is necessarily a multiple of three nucleotides. Therefore, should a stretch of DNA start and end with stop codons (TAA, TAG, TGA) separated by a whole number of nucleotide triplets, the question arises as to whether this DNA stretch is coding or not. Hereafter, we will refer to these DNA stretches as “open reading frames” (ORF).

ORFs are expected to be shorter in DNA sequences with AT (Adenine + Thymine) levels >50% for the obvious reason that A and T are more frequent in stop codons than G. Since there are three stop codons and 61 amino acid codons, (3:61) a stop codon occurs with a probability of approximately one in twenty (1:20). Furthermore, given three base pairs per codon, this should lead to one stop codon every sixty base pairs, in which A, C, G or T are equally likely to occur. Therefore, one would expect the ORF size to be around 60 bp. Of course, the frequency of stop codons may vary significantly depending upon the local nucleotide composition (see below in the section of Results). However, one could say that the probability of an ORF being a coding sequence increases with its size. Most proteins are larger than 100 codons (300 bp) and their ORFs should be, therefore, relatively easy to classify. Unfortunately, the coding sequences (CDS) of eukaryotes are split up by the non-coding DNA of introns leaving coding stretches (exons) <300 bp.

The physico-chemical constraints on proteins induce specific usage of nucleic triplets that can be efficiently detected by Markov Models.^1^ Investigating the evolutionary origin of the genetic code, Ikehara et al^2^ showed that it may have originated from a four-amino acid system, the GNC code. This GNC code (G for Guanine, N for any of the 4 nucleotides, C for Cytosine) is able to encode GADV-proteins (G for Glycine, A for Alanine, D for Aspartic acid, V for Valine) with appropriate three-dimensional structures, being water soluble globular proteins (hydropathy, α-helix, β-sheet, and β-turn) and also having catalytic activities.^3^ According to Ikehara et al,^2^ this primitive code would have evolved first in a code with 16 codons and ten amino acids, the so called SNS (S for strong: G or C) and then in the RNY (R for purines, Y for pyrimidines) ancestral codon suggested by Shepherd.^4^ Consequently, the coding DNA is characterized by at least two fundamental features: (i) the absence of the in-frame stop codon and (ii) a higher purine frequency in 1st position of codons^4^ that we called the ‘purine bias’ (Rrr).

Next, we investigated the contribution both of Rrr bias and also of stop codon distribution in the classification of coding vs non-coding ORFs. Our methodology is designed for the diagnosis of coding ORFs in small DNA sequences in the size range 200 to 1000 bp with the assumption that they contain a single coding region. Larger sequences where multiple coding regions are expected would need to be investigated with a sliding window. The procedure involves four steps: (i) Extracting all ORFs from the six frames of a given DNA fragment. (ii) Attributing a putative coding strand to these ORFs. (iii) Eliminating those ORFs without the purine bias of CDSs. (iv) Selecting the largest of these ORFs and declaring it as CDS. To eliminate false positives due to very small ORFs, we filtered them out by setting a minimal size threshold. Consequently, ORFs are simply classified as non-coding when they do not match the Rrr bias above a given size threshold.

Exploring CDSs and introns among six model species covering the whole spectrum of codon usage in eukaryotes, we found that the strand diagnosis is >95% at 350 bp and that the success rate of the coding diagnosis is >98%. However, we found that <18% of the CDSs whose size is 350 bp may not be detected. Tightening up our classification for “true” coding DNA is possible, but would affect the number of ORFs effectively retrieved.

## Materials and Methods

### Coding features

We revisited the contribution of purines to coding sequences (CDS) by computing the relative frequency of the four nucleotides Adenine, Cytosine, Guanine and Thymine (A, C, G and T) in the three positions of triplets and the six frames (the three frames on both plus and minus strands). All relative frequencies of this study were calculated as the ratio of a given occurrence to the number of contiguous triplet *N* = *n*/3 where *n* is the nucleotide number in the sequence. The relative nucleotide frequencies were denoted *P_i_* with *i* ∈ {A, C, G, T}. The contribution of purines (A and G) was evaluated in the three positions *j* ∈ {1, 2, 3} of triplets by computing both the sum (*P*_*A*1_ + *P*_*G*1_, + *P*_*A*2_ + *P*_*G*2_, *P*_*A*3_ + *P*_*G*3_ that we noted AG1, AG2, AG3, respectively, in the following) and the product (*P*_*A*1_*P*_*G*1_, *P*_*A*2_*P*_*G*2_, *P*_*A*3_*P*_*G*3_) of their relative frequencies over the six frames *k* ∈ {−1, −2, −3, +1, +2, +3}. We also computed the relative frequency of stop codons TAA, TAG, TGA (*P_STOP_*) and the product of relative frequencies of C, G and A in the three consecutive positions of triplets, i.e. (*P*_*C*1_*P*_*G*2_*P*_*A*3_), (*P*_*G*1_*P*_*A*2_*P*_*C*3_), and (*P*_*A*1_*P*_*C*2_*P*_*G*3_), over the six frames.

Using the frequencies just described, we set up five features for the diagnosis of coding ORFs as follows: (i) The quantity *f*_1_ = 1− *P_STOP_*. If we consider the example of a coding sequence, *f*_1_ is equal to 1 in frame *k =* +1 since there is no in-frame stop codon within the coding frame of a coding sequences and since we defined the ORF as a DNA stretch between two stop codons separated by a whole number of nucleotide triplets, or alternatively as a DNA stretch between a sequence extremity and a stop codon separated by a whole number of nucleotide triplets. By contrast, *f*_1_ is expected ≤1 in non-coding frames because there is no constraint against stop codons in these frames. The value of *f*_1_ in non-coding frames is expected to decrease with the size of the coding sequence at a rate that is proportional to its AT level. (ii) We also found that the statements *P*_*C*1_*P*_*G*2_*P*_*A*3_ < *P*_*G*1_*P*_*A*2_*P*_*C*3_ and *P*_*C*1_*P*_*G*2_*P*_*A*3_ < *P*_*A*1_*P*_*C*2_*P*_*G*3_ are generally true (93% of the cases) in frame *k* = +1. Therefore, the features *f*_2_ = 1−*P*_*C*1_*P*_*G*2_*P*_*A*3_ and *f*_3_ = *P*_*G*1_*P*_*A*2_*P*_*C*3_ − *P*_*C*1_*P*_*G*2_*P*_*A*3_ + *P*_*A*1_*P*_*C*2_*P*_*G*3_ − *P*_*C*1_*P*_*G*2_*P*_*A*3_ are also positive and maximum in most coding frames (see below). (iii) As stated above, the coding sequences are characterized by a purines bias.^4^ Therefore, one has *P*_*A*1_*P*_*G*1_ > *P*_*A*2_*P*_*G*2_ and *P*_*A*1_*P*_*G*1_ > *P*_*A*3_*P*_*G*3_ in frame *k* =+ 1 and the quantity *f*_4_ = *P*_*A*1_*P*_*G*1_ − *P*_*A*2_*P*_*G*2_ + *P*_*A*1_*P*_*G*1_ − *P*_*A*3_*P*_*G*3_ should be positive and have its maximum in frame *k* = +1. (iv) A significant proportion of GC-rich CDSs are deprived of a stop codon on more than one frame over large sequence sizes (>300 bp). However, most CDSs with GC > 55% are also *P*_*G*1_*P*_*C*1_ > *P*_*G*2_*P*_*C*2_ (see below). We took this into account by calculating the feature *f*_5_ = *P*_*G*1_*P*_*C*1_ − *P*_*G*2_*P*_*C*2_.

The procedure of coding ORF diagnosis described here involves the following steps: (i) the diagnosis of the coding strand, (ii) the identification of the ORFs that have a purine bias similar to that of CDSs and (iii) the extraction of the largest of these putative coding ORFs.

### Strand classification

We tested the success rate of coding strand classification on the 5′ and 3′ sides of CDSs. For this, we extracted sequence pieces whose sizes varied between 50 and 600 bp from both CDS extremities. We then calculated the quantity *S* = *f*_1_ + *f*_2_ for all ORFs over the six frames of each of these CDS pieces. The sequences corresponding to frames *k* = −1, −2, −3 (the minus strand) were converted in their equivalent *k* = +1, +2, +3 in order to evaluate all sequences in their 5′−3′ orientation. An ORF from the plus strand was considered potentially coding when the maximum of *S* was found for a frame of the plus strand, i.e. frames +1, +2, +3. Similarly, an ORF from the minus strand was considered potentially coding when the maximum of *S* was found for a frame of the minus strand, i.e. frames −1, −2, −3. When an ORF from the plus strand corresponded to the maximum of *S* for a frame of the minus strand, i.e. −1, −2, −3, and *vice versa*, the ORF was eliminated from the list.

### Coding vs. non-coding classification

The ORFs selected as described above must then be confirmed for their coding potential. We classified a sequence as coding or non-coding (intron) by scoring the purine bias. For this, we calculated the maximum of the quantity *C* = *f*_1_ + *f*_3_ + *f*_4_ + over the six frames *k*. When *C* was higher than a threshold the sequence was classified coding, when lower, non-coding. The threshold value was found to be 1.05.

We slightly improved the success rate of *C* in GC-rich sequences by calculating the maximum of the quantity *C* = *f*_1_ + *f*_3_ + *f*_4_ + *f*_5_ over the six frames when the GC level of the sequence was >55%, otherwise we calculated the maximum of the quantity *C* = *f*_1_ + *f*_3_ + *f*_4_, as described above.

### Minimum ORF size for coding diagnosis

Considering a DNA sequence, its largest ORF (LORF) is not necessarily the coding one. For instance, considering the sequence of an expressed sequence tag (EST) from the 3′ end of a cDNA, an ORF in the 3′ UTR (non-coding) can be larger than the piece of coding sequence that it contains. However, the largest ORF among the ORFs that are classified coding (LcORF) has a higher probability of being actually coding. Here, we consider “coding” ORFs to be those with the Rrr bias of CDSs. Thus, LcORF, (i.e. the largest of the ORFs with Rrr bias) has higher probably to represent the actual coding ORF of a DNA segment. ORFs containing around 150 to 200 bp with Rrr bias are relatively common in introns. Intronic LcORFs are therefore a potential source of false positives. We investigated their size distribution in comparison to that of LORFs among the six frames of introns. The comparison of LORF and LcORF distributions is informative concerning the gain in sensitivity that is achieved by taking the Rrr bias into account for coding ORF diagnosis. Of course, the strategy of selecting the LcORF as the only coding ORF candidate eliminates the possibility of detecting coding ORFs that would overlap on the plus and minus strand. This has been done deliberately to simplify the experimentation and does not alter our conclusions.

### Algorithm

The procedure outlined above can be summarized in the following algorithm:

1. Load the sequence,
2. Scan the three frames in the “+” and “−” (the complementary) strands,
3. Construct a table with the ORFs of the three frames by splitting the corresponding sequence according to stop codons for “+” e “−” strands,
4. For each strand, scan the ORF table and:
  - measure the ORF size,
  - if the ORF is larger than the selected size threshold:
    ○ calculate the *f*_1_, *f*_2_, *f*_3_, *f*_4_, and *f*_5_ in the six frames of the ORF under analysis,
    ○ search among the six frames the one that corresponds to the maximum of *S*,
    ○ if the maximum occur for a frame ≤3, the strand is declared “+”,
    ○ continue if the strand is declared “+”, otherwise analyze the next ORF,
    ○ if GC_ORF_ < 55%
      ▪ if *C*_1_ ≥ 1.05, the ORF is declared “coding”,
    ○ if GC_ORF_ ≥ 55%
      ▪ if *C*_2_ ≥ 1.05, the ORF is declared “coding”,
5. Chose the largest (LcORF) among “+” and “−” ORFs.

### Sequence material

Given that this study tends to be a reference case, we built datasets with CDSs of six model species covering the complete range of GC levels in 3rd positions of codons (GC3) and sequence complexity in eukaryotes. We chose GC3 as a criterion to evaluate codon usage diversity. Because of degeneration in the genetic code affecting 3rd position of codons, it is here that both variation in GC and also codon usage is the most extensive. Codon usage has been proven to interfere with the efficiency of gene prediction. It is the main factor explaining why algorithms based on machine learning must be trained. Therefore, a fundamental issue in gene prediction concerns the degree of codon variation which exists between species, as seen in these reference sequences. To avoid interferences with false positives of predicted genes, we filtered out the CDSs that were not experimentally validated through a peer reviewed publication in order to avoid the possible contribution of annotation errors.

Among the species considered here, *Plasmodium falciparum* (CDS= 197, GC3 = 0%–30%) is extremely GC-poor^5^ while *Chlamydomonas reinhardtii* (CDS= 102, GC3 = 60%–100%) is extremely GC-rich.^6^ These two species stand at opposite ends of the spectrum of eukaryote GC3 variation. *Arabidopsis thaliana* (CDS = 1,206, GC3 = 25%–65%) has a genome whose GC level^7^ is representative of core dicots^8^ while *Oryza sativa* (CDS = 401, GC3 = 25%–100%) is a species representative of *Gramineae*. The common ancestor of this plant family underwent a transition of nucleotide composition.^8^,^9^ The consequence of this transition is that the genes of this species are shared in two classes with two different codon usages. This feature confounds gene prediction in this species.^9^,^10^ *D. melanogaster* (CDS = 1,262, GC3 = 40%–85%) is a species that also underwent a transition of nucleotide composition among insects.^11^ Finally, *H. sapiens* (CDS = 1,199, GC3 = 30%–90%) is representative of warm-blooded vertebrates.^12^ Because of the transition of nucleotide composition that occurred in mammals, the genes of *H. sapiens* are shared in five different classes.^13^ Another important sequence feature for the purpose of gene prediction is sequence entropy^14^ since its increase may lead to decrease the level of the Rrr bias.

Complete nuclear CDSs of the above species were retrieved from GenBank (release 137, August 15th, 2003) and filtered according to Carels et al^9^ in order to eliminate redundancy and potentially false positive or doubtful genes resulting from wrong *in silico* predictions. The sequences all started with ATG and ended with a stop codon and none included in-frame internal stop codon.

We also built datasets of CDS fragments (frame + 1) of the six model species with fixed sizes of 50, 100, 150, 200, 250, 300, 350, 400, 450, 500, 550, 600 bp extending from (i) the first ATG until the desired sequence size and from (ii) the 3′ side (next to the stop codon, but excluding it).

We tested the success rate of exon/intron classification with the CDS samples just described and the samples of intron sequences of *A. thaliana* (n = 5,301), *D. melanogaster* (n = 18,749), *H. sapiens* (n = 2,030) retrieved from http://hsc.utoledo.edu/bioinfo/eid/index.html. Intron datasets were built by cutting pieces of fixed size of 50, 100, 150, 200, 250, 300, 350, 400, 450, 500, 550, 600 bp extending from the 5′ side to the desired sequence size.

## Results

According to Shepherd,^4^ we found that the purine level is the highest, on average, in the 1st position of codons of all six species (data not shown). Therefore, we denoted this purine bias by Rrr. However, the difference between the product of purine probabilities in 1st and 2nd positions was higher than that between the sum (%) of these probabilities.

**Figure 1.**
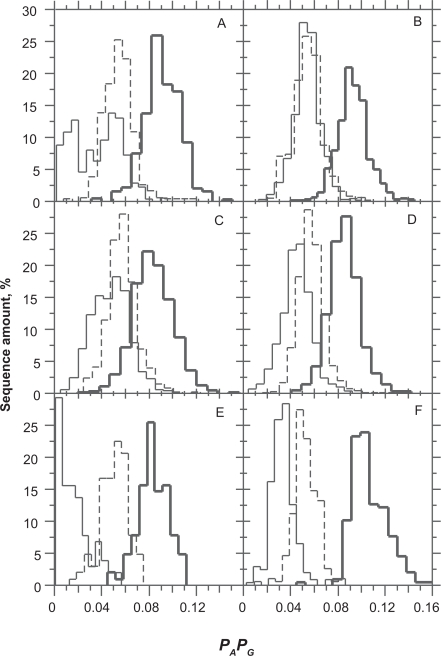
Distribution of the product of purines (**A**, **G**) probabilities (*P_A_P_B_*) in *O. sativa* (**A**), *A. thaliana* (**B**), *H. sapiens* (**C**), *D. melanogaster* (**D**), *C. reinhardtii* (**E**) and *P. falciparum* (**F**). The product of purine probabilities is higher, on average, in the 1st position of codons (bold) than in the 2nd (dashed) and in the 3rd (thin).

The product of purine probabilities was, on average, *P*_*A*1_*P*_*G*1_ = 0.09 and *P*_*A*2_*P*_*G*2_ = 0.05. Both values are remarkably conserved among distant species whatever their average GC level (Fig. 1). Two peaks of purine distribution in 3rd position of codons were found for rice (Fig. 1A). One, centered on *P*_*A*3_*P*_*G*3_ = 0.015, is characteristic of extremely GC-rich genomes such as *C. reinhardtii* (Fig. 1E). The second peak centered on *P*_*A*3_*P*_*G*3_ = 0.050, is common to the other genomes (Table 1). Table 1 shows that the product of purine probabilities in 3rd codon position is close to 0 for extremely GC-rich CDSs.

Despite its extremely high AT composition, *P. falciparum* also shows the Rrr bias (Fig. 1F). The Rrr bias promotes purine compensation between the three positions of codons (Table 2). The intensity of these compensations changes according to the species. It is interesting to note that in contrast to A, G does not show correlation between 1st and 2nd positions of codons in any of the six species.

**Figure 2.**
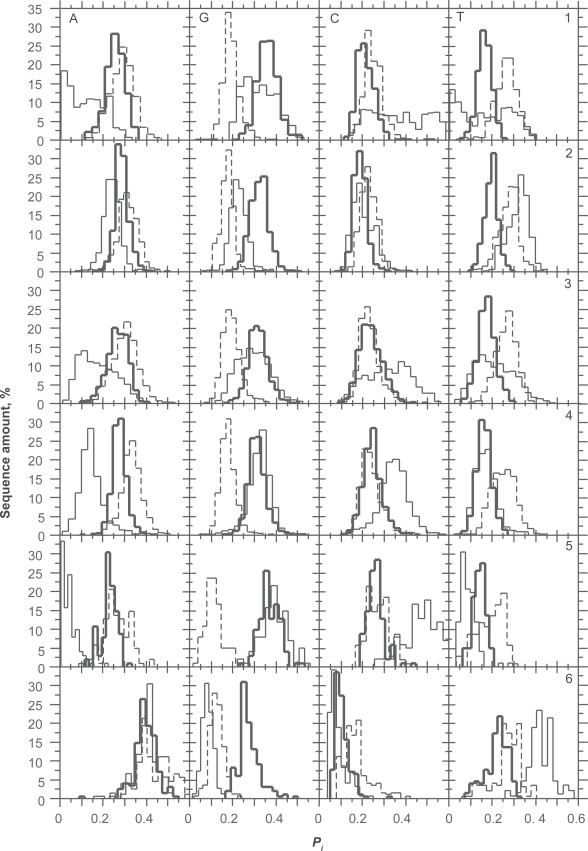
Distribution of nucleotide probabilities (**A, G, C, T**) in 1st (bold), 2nd (dashed) and 3rd (thin) positions of codons in *O. sativa* (1), *A. thaliana* (2), *H. sapiens* (3), *D. melanogaster* (4), *C. reinhardtii* (5) and *P. falciparum* (6).

**Figure 3.**
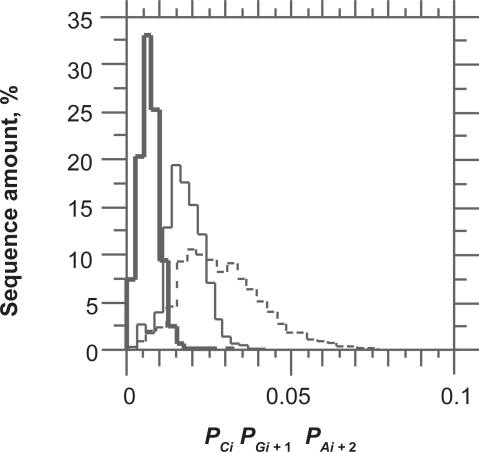
Distribution of *P*_*C*1_*P*_*G*2_*P*_*A*3_ (bold), *P*_*G*1_*P*_*A*2_*P*_*C*3_ (dashed) and *P*_*A*1_*P*_*C*2_*P*_*G*3_ (thin) in the coding sequences of *O. sativa*, *A. thaliana*, *H. sapiens*, *D. melanogaster*, *C. reinhardtii* and *P. falciparum* grouped together.

In agreement to Figure 2, *P*_*A*1_ and *P*_*G*1_ are relatively constant over species except in *P. falciparum* where both purines obviously compensate each other. The absence of correlation between *P*_*A*1_ and *P*_*G*1_ in *H.sapiens* and *D. melanogaster* (Table 2) is not surprising since their distributions overlap closely. The correlation between *P*_*A*1_ and *P*_*A*2_ is more surprising since they also overlap closely. This shows that the correlation can be significant over a very small range of variation in base composition. By contrast, the absence of correlation between *P*_*G*1_ and *P*_*G*2_ is surprising since the relationship between these two bases is such that *P*_*G*2_ is lower than *P*_*G*1_ in every species. The difference between *P*_*G*1_ and *P*_*G*2_ is larger than that between *P*_*A*1_ and *P*_*A*2_ (Fig. 2). We also found negative correlations between *P*_*A*1_*P*_*G*1_ and GC3 (−0.37), on the one hand, and between AG1 and GC3 (−0.35), on the other hand. The major contribution to these correlations is due to A1 since the correlation between *P*_*A*1_ and GC3 was −0.57 while that between *P*_*G*1_ and GC3 was 0.20. The negative correlation of purines in 1st position of codons and GC3 shows that the purine bias Rrr tends to be weaker for GC-rich genes. Other interesting regularities that can be derived from Figure 2 are that *P*_*C*1_, *P*_*G*2_ and *P*_*A*3_ are lower than their respective probabilities in other positions of codons. A3 is clearly compensated by C3 as appears from negative correlation between A3 and C3 (r = −0.9, data not shown). This is shown at Figure 3 where the overlap between *P*_*C*1_*P*_*G*2_*P*_*A*3_, *P*_*G*1_*P*_*A*2_*P*_*C*3_ and *P*_*A*1_*P*_*C*2_*P*_*G*3_ is only 7% of the CDSs of the six species considered together. This property of CDS is the consequence of the Rrr bias. It is essential for the diagnosis of the coding strand in GC-rich sequences. However, it must be used in combination with stop codon distribution to allow sufficient success rate (see below).

**Figure 4.**
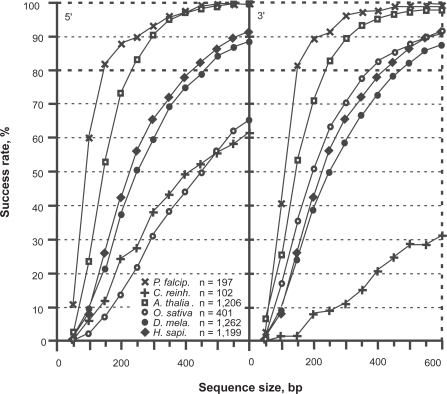
Classification of the coding frame among the six frames of coding sequences between 50 and 600 bp. The success rate of *S* = *f*_1_ is shown for *P. falciparum* (X), *C. reinhardtii* (+), *A. thaliana* (□), *O. sativa* (O), *D. melanogaster* (•) and *H. sapiens* (♦), respectively.

**Figure 5.**
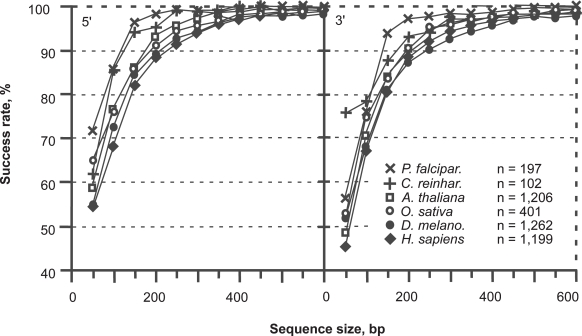
Classification of the coding frame among the six frames of coding sequences between 50 and 600 bp. The success rate of the function *S* = *f*_1_ + *f*_2_ over six frames is shown for *P. falciparum* (X), *C. reinhardtii* (+), *A. thaliana* (□), *O. sativa* (O), *D. melanogaster* (•) and *H. sapiens* (♦), respectively.

The bias in stop codon distribution introduced by the coding frame is not satisfactory for a secure diagnosis of the coding strand when GC-rich CDSs are small (Fig. 4). The success rate of coding strand diagnosis using stop codons only depends on the average level of AT. Short GC-rich sequences (*O. sativa* and *C. reinhardtii*) can be deprived of stop codon in non-coding frames as well. Therefore, the quantity *S* = *f*_1_ + *f*_2_ allows much more accurate coding strand diagnosis *S* = *f*_1_ (Fig. 5).

**Figure 6.**
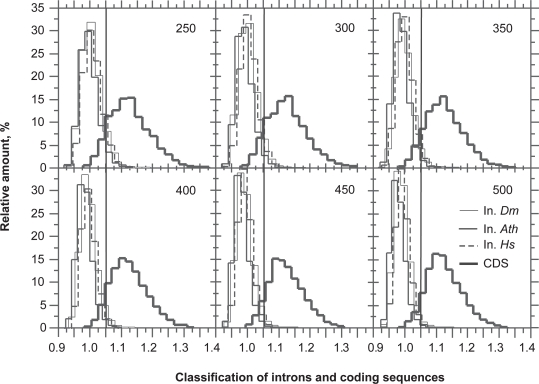
Classification of coding sequences (CDS) and introns (In) between 250 and 500 bp and among the six frames. The intron distributions of *A. thaliana* (*Ath*, plain), *D. melanogaster* (*Dm*, thin) and *H. sapiens* (*Hs*, dashed) are centered on the classification value of 0.95. The CDS distribution of the six species grouped together (bold) are centered on the classification value of 1.10. The plain line (vertical) is for the threshold of classification of introns and CDSs at 1.05. The classification function was *C* = *f*_1_ + *f*_3_ + *f*_4_ below GC = 55% and *C* = *f*_1_ + *f*_3_ + *f*_4_ + *f*_5_ above GC = 55%.

**Figure 7.**
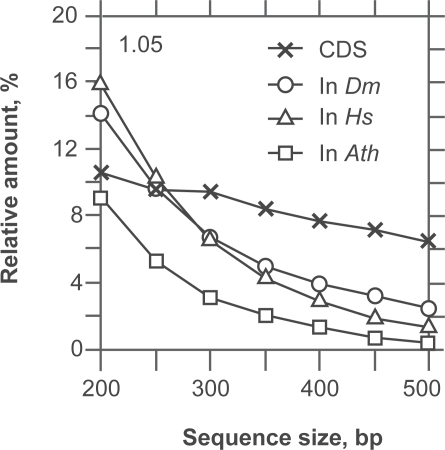
Relationship between false positives (In) and false negatives (CDS) at sequence sizes between 200 and 500 bp for the thresholds of classification at 1.05. The introns (In) in this plot are from *A. thaliana* (□), *D. melanogaster* (O) and *H. sapiens* (Δ). The introns indicate the proportion of false positives because they are classified as coding while they are not. The coding sequences (X) are from the six species of Figure 6 grouped together. They indicate the proportion of false negatives because they are classified as non-coding while in fact they are.

However, the power of this simple function for the classification of exons and introns is low (data not shown). We found a solution to this problem by measuring the asymmetry introduced by the Rrr bias. The asymmetry of GC-poor CDSs (GC < 55%) can be scored with the quantity *C* = *f*_1_ + *f*_3_ + *f*_4_. When CDSs are GC-rich (GC > 55%) as occurs in *O. sativa* and *C.reinhardtii*, a success rate higher by 4%–5% (data not shown) is obtained with the quantity *C* = *f*_1_ + *f*_3_ + *f*_4_ + *f*_5_ (Figs. 6, 7). Figure 6 shows the performance of the classification of introns and CDSs with increasing sequence size. Three different intron sources were plotted in Figure 6: *A. thaliana*, *H. sapiens* and *D. melanogaster*. The intron distribution of *A. thaliana* is the most homogeneous among the three and, therefore, *A. thaliana* is the species with the highest success rate of intron/exon classification among the three species tested. For the purpose of clarity, we group the CDSs of the six species all together. The overlapping area (Fig. 6) concerns the sequences for which the intron/exon classification cannot be trusted. The classification threshold can be chosen according to two strategies: optimize the error rate or maximize true positives. Considering Figure 6, the plain vertical line is for the threshold at 1.05 (see also Fig. 7). With a threshold of 1.05, the proportion of exons that are classified as introns (false negatives) is 10% at 200 bp and 7% at 600 bp. On the other hand, the proportion of introns that are classified as exons (false positives) is between 8% (*A. thaliana*) and ~15% (*H. sapiens*, *D. melanogaster*) at 200 bp and between 0 and 3% at 600 bp (Fig. 7). The error due to false positives decreases more rapidly than that due to false negatives.

**Figure 8.**
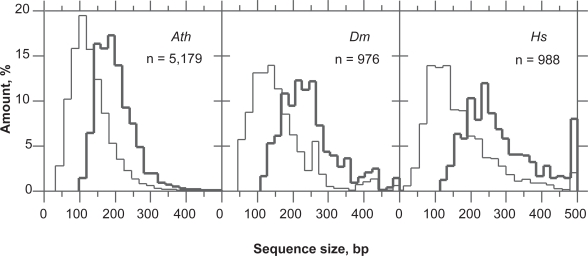
Distribution of ORF size in introns of *A. thaliana* (*Ath*), *D. melanogaster* (*Dm*) and *H. sapiens* (*Hs*). The largest ORF (bold line) is a reference for the largest ORF that matches the purine bias of a coding sequence (thin line). The distance between the peaks of both distributions measures the gain of introducing the Rrr scoring for coding ORF diagnosis. It also shows the limit of resolution of exon/intron classification with this method.

We found that the largest ORF (LORF) in introns of *A. thaliana*, *D. melanogaster* and *H. sapiens* are between 200 and 250 bp, on average (Fig. 8). The distribution of the largest ORFs showing the purine bias (LcORF) peaks at 100 bp in all three species and trails off towards ~300 bp in *Arabidopsis* and *Drosophila*. In humans, the LcORF distribution trails until ~400 bp (the bar at 500 bp in the LORF distribution most probably indicating the dataset contamination by CDSs. According to this speculation, the contamination rate could be as high as 8%). If we consider 2.5% as an acceptable rate of false positives in intron/exon classification, LcORFs from *A. thaliana* can be considered coding in 97% of the cases provided that they are >300 bp (Fig. 8). The size threshold for LcORFs of *A. thaliana* under the success rate of 95% is ~240 bp, which results in a gain of ~60 bp in sensitivity. According to the same criteria, the size threshold above which LcORF classification is reached with a 95% success rate is (i) between 150 and 200 bp for *P. falciparum* and *C. reinhardtii*, (ii) 300 bp for *D. melanogaster* and (iii) 350 bp for *H. sapiens*.

## Discussion

The methodology presented here is an attempt to understand the features of coding sequences that allow their classification independently of the species.

We investigated a set of model species that cover the entire range of codon usage and sequence complexity in eukaryotes. The unicellular *Plasmodium falciparum* is extremely rich in AT while *Chlamydomonas reinhardtii* is, by contrast, extremely rich in GC. This warrants the coverage of the complete codon usage. *Arabidopsis thaliana* has an average base composition that is representative of the dicots and monocot plant species. Rice is representative of the *Gramineae* family that has the particularity of having two gene classes one with a codon usage typical of angiosperms in general and one that is extremely GC-rich as in *C. reinhardtii*.^6^ *Drosophila melanogaster* and *Homo sapiens* are two species that demonstrate a compositional transition in their respective common ancestor.^11^,^12^ For this reason, they are expected to be more heterogeneous in their sequences.

Despite the enormous genetic distance between these species, we found a common model for their coding sequence (CDS). The model is based on the stop codon distribution and on the purine bias (Rrr) in CDSs. The purine bias has been claimed to be a universal feature of CDSs^4^ that could help to classify them in the process of gene finding. However, the purine bias has also the corollary that *P*_*C*1_ *P*_*G*2_ *P*_*A*3_ reaches its minimum value in the coding frame of CDSs. As far as we know, this feature has not been described before, but it is essential for the successful diagnosis of CDSs using the purine bias as proposed by Shepherd.^4^ The *P*_*C*1_*P*_*G*2_*P*_*A*3_ bias results from the nucleotide compensations that occur in the CDSs with the effect of generating a higher abundance of purine in 1st position of codons than in the two other positions (Rrr). The compensation occurs in such a way that A1 is more abundant in AT-rich (*P. falciparum*) and G1 is more abundant in GC-rich (*C. reinhardtii*) genomes. This is obvious from the negative correlation (−0.57) between A1 and GC3 and from the positive correlation between G1 and GC3 (0.20). However, whether AT-rich or GC-rich, G is more abundant in 1st than in 2nd position of codons.^2^ This can be regarded as a remnant of the GNC ancestral codon.^2^ This feature is essential since it is conserved in *P. falciparum*. However, in the particular case of this species a substantial number of codons take A1 in place of G1. The absence of correlation between *P*_*G*1_ and *P*_*G*2_ by contrast to the correlation between (i) *P*_*A*1_ *and P*_*A*2_ and (ii) *P*_*A*1_ *P*_*G*1_ and *P*_*A*2_*P*_*G*2_ suggests that different constraints act on A and G. Reasons for this can be found in the *universal correlation*.^15^

Actually, Rrr is a feature that allows the measure of codon asymmetry in CDSs as does the CSF function.^16^ The reason for codon asymmetry in CDSs is not trivial. There is the same number of RNN and YNN codons in the genetic code. The larger frequency of Rrr in CDSs is due to the proteomic code. To sum up, it is the consequence of constraints acting on secondary and 3D protein structures.^17^

When used alone, the purine bias Rrr allows coding frame detection with only ~84% success rate (data not shown). The most important source of frame confusion is from frame −1. An explanation for this is found in Biro’s review.^17^ Complementary codons often encode complementary amino-acids that are involved in 3D protein folding. The balance of sense and antisense codons is close to the equilibrium, which justifies an error rate of ~15% on the coding frame diagnosis by Rrr. For this reason, Rrr should be used only for the coding diagnosis and not for the strand diagnosis.

In AT-rich sequences, the bias of stop codon(s) distribution among frames is sufficient to allow the elimination of most frame ambiguities in sequences >350 bp. In GC-rich sequences (>0.55% GC), the introduction of the condition *P*_*G*1_*P*_*C*1_ > *P*_*G*2_*P*_*C*2_ in combination to the *P*_*C*1_*P*_*G*2_*P*_*A*3_ and stop codon(s) biases is necessary. The probability of stop codons is too low in GC-rich ORFs ~350 bp to allow unambiguous frame diagnosis. Fortunately, *P*_*C*1_*P*_*G*2_*P*_*A*3_ compensates for this lack of specificity. In addition, the condition *P*_*G*1_*P*_*C*1_ > *P*_*G*2_*P*_*C*2_ combined with the conditions *P*_*A*1_*P*_*G*1_ > *P*_*A*2_*P*_*G*2_ and *P*_*A*1_*P*_*G*1_ > *P*_*A*3_*P*_*G*3_ compensates for the negative correlation between A1 and GC3 with the consequence that the success rate of exon/intron classification remains at a high level.

The purine bias induced by the physico-chemical properties of proteins is sufficient to classify CDSs from introns with a success rate >95% above 350 bp. The threshold of >95% success rate is found at lower ORF size in AT-rich sequences. This suggests a positive correlation between the exon size and their GC level. This correlation has been detected in plants^18^ and vertebrates.^19^

The different success rates of exon/intron classification between *A. thaliana*, on one hand, and *H. sapiens*, *D. melanogaster*, on the other hand, are apparently due to intrinsic difference of base composition. The difference of GC level between introns and exons was found to be higher, on average, in *A. thaliana* (5% to 15%–30%),^7^ than in *H. sapiens*,^20^ *D. melanogaster* (5%). In addition, the vast majority of plant introns are GC-poor,^18^ which is not the case in *H. sapiens* and *D. melanogaster*.

The features analyzed in this study allow an improvement to the sensitivity of exon vs intron classification by 50 to 150 bp at small ORF sizes compared to other methods, i.e. the Average Mutual Information from Grosse et al^14^ and the CSF function from Nikolaou and Almirantis,^16^ which claim to be independent of codon usage, and which do not need a training step. However, the substantial difference is that these aforementioned methods predict neither the strand nor the coding frame. In consequence, we believe that our method could be helpful in the extraction of coding ORFs from ESTs and/or from metagenomic reads. It could also help in the preparation of training set for *ab initio* gene prediction with machine learning algorithms in those genomes for which little information is available.

## Figures and Tables

**Table 1.** Product of purine probabilities in the three positions of codons.

**Species**	***Sz*^1^**	***P*_*A*1_*P*_*G*1_**	***σ*_*A*1*G*1_^2^**	***P*_*A*2_*P*_*G*2_**	***σ*_*A*2*G*2_**	***P*_*A*3_*P*_*G*3_**	***σ*_*A*3*G*3_**	**Δ_*AG*1, 2_^3^**	**Δ_*AG*2, 3_**
*O. sativa*	401	0.091	0.016	0.054	0.012	0.036	0.020	0.037	0.018
*GC-poor*	227	0.095	0.014	0.055	0.012	0.050	0.013	0.040	0.005
*GC-rich*	174	0.086	0.016	0.054	0.013	**0.018**	**0.012**	**0.032**	**0.036**
*A. thaliana*	1206	0.093	0.013	0.055	0.013	0.055	0.011	0.038	0.000
*H. sapiens*	1199	0.084	0.017	0.058	0.013	0.048	0.015	0.026	0.010
*D. melanogaster*	1262	0.086	0.013	0.058	0.012	0.045	0.013	0.028	0.013
*C. reinhardtii*	102	0.084	0.013	0.051	0.012	**0.017**	**0.013**	**0.033**	**0.034**
*P. falciparum*	197	0.107	0.017	0.052	0.010	0.033	0.010	0.055	0.019

1 *Sz* is the sample size of coding sequences.

2 σ is the standard deviation for the product of probabilities of the nucleotide pair under consideration.

3 Δ is the difference of σ between two positions of codons.

**Table 2.** Correlations between purine probabilities at one or two position(s) of codons.

**Species**	***Sz*^1^**	***P*_*A*1_,*P*_*A*2_**	***P*_*A*1_,*P*_*A*3_**	***P*_*G*1_,*P*_*G*2_**	***P*_*G*1_,*P*_*G*3_**	***P*_*A*1_,*P*_*G*1_**	***P*_*A*2_,*P*_*G*2_**	***P*_*A*3_,*P*_*G*3_**	***P*_*A*1_,*P*_*G*2_**	***P*_*A*1_,*P*_*G*3_**
*O. sativa*	401	0.44^2^	0.45	0.21	0.27	−0.50	−0.38	−0.74	−0.35	−0.43
*A. thaliana*	1206	0.43	0.16	0.12	0.11	−0.40	−0.26	−0.22	−0.10	−0.10
*H. sapiens*	1199	0.44	0.51	0.17	0.23	−0.35	−0.47	−0.80	−0.50	−0.50
*D. melanogaster*	1262	0.13	0.32	0.00	0.10	−0.34	−0.40	−0.68	−0.18	−0.29
*C. reinhardtii*	102	0.30	−0.30	0.30	0.33	−0.49	−0.32	0.04	−0.24	−0.19
*P. falciparum*	197	0.52	−0.06	0.10	−0.22	−0.59	−0.71	−0.16	−0.26	0.14

1 *Sz* is the sample size of coding sequences.

2 All the values >0.20 or <−0.20 were statistically significant at *P* < 0.001. The values >0.40 were placed on gray background to facilitate table analysis.
